# Communicating cancer to children: Strategies and needs of parents with cancer. A qualitative study

**DOI:** 10.1017/S1478951525101272

**Published:** 2026-01-08

**Authors:** Sara Alfieri, Bianca Scacciati, Zaira Nardone, Marco Romeo, Luminita Andreescu, Mariangela Chiorazzi, Pauline Dimastromatteo, Elena Burattini, Rossana Berardi, Simonetta Zappata, Valentina Belbusti, Laura Gangeri, Claudia Borreani

**Affiliations:** 1 Clinical Psychology Unit, Fondazione IRCCS Istituto Nazionale dei Tumori, Milan, Italy; 2 ANVOLT OdV, Milan, Italy; 3 ANVOLT OdV, Fano, Italy; 4 Clinica Oncologica, Università Politecnica delle Marche, Azienda Ospedaliero Universitaria delle Marche, Italy; 5 Palliative Care, Pain Therapy and Rehabilitation Unit, Fondazione IRCCS Istituto Nazionale dei Tumori, Milan, Italy; 6 Semper Onlus OdV, Fano, Italy

**Keywords:** Cancer, communication, minor children, parenting, psycho-oncology

## Abstract

**Objectives:**

Communicating a cancer diagnosis to a child is a complex challenge for parents. This study aims to explore (1) the communication strategies and beliefs of parents with cancer when communicating with their children and (2) the needs of these parents.

**Methods:**

Semi-structured interviews were conducted with parents with cancer being treated at an Italian comprehensive cancer center and their healthy partners, when present. The interviews were analyzed through a constructivist approach using reflexive thematic analysis. The number of parents to be interviewed was not predetermined, but the meaning saturation procedure was followed.

**Results:**

Ten parents were interviewed, meaning saturation was reached at the seventh interview. Five themes were created: (1) the challenges parents faced at this sensitive time; (2) the emotions parents experienced; (3) the beliefs that may have influenced how they communicate the illness to their children; (4) the strategies parents used to communicate the illness to their children and (5) parents’ perception of their children’s understanding of the illness. Fifty-seven needs, often unmet, were also identified and were grouped into three categories: (1) “existential” needs; (2) support needs; and (3) needs related to continuing to be and act as parents.

**Significance of results:**

This study provides important insights for healthcare professionals to consider in order to better support and care for these parents.

## Introduction

The family is a dynamic social organization of people “in relationship” with each other, based on the interdependence of its members (Cooley 1909; Scabini and Iafrate 2019). Because of this particular configuration, the diagnosis of cancer in a parent doesn’t just affect the individual person, but the whole family organization.

Parents who discover having cancer may experience a loss of energy, physical fatigue, anxiety and worry; they also have to undergo repeated visits and tests, which inevitably disrupt the daily routine of the whole family (Semple and McCance 2010). To these stressors should be added the worry and exhaustion of continuing to fulfill their fundamental role as parents (Zaider et al. 2015; Check et al. 2017; Morris et al. 2018).

One of the most difficult aspects for parents is how and what to tell their children about cancer (Semple and McCance 2010; Schiena et al. 2019). Some noteworthy systematic reviews (Semple and McCance 2010; Xu et al. 2024) have collected studies (mainly qualitative) on the strategies used by parents with cancer to communicate the illness to their children. The results show that although parents favor open communication with their children, they find it difficult to put this into practice, mainly because they do not feel adequately prepared. This outcome aligns with the recent systematic review by Mckeown et al. (2025), which highlighted how children may experience isolation at home due to constrained family communication. The authors indicate that lack of parental responds to young people’s informational needs lead to rumination and a sense of loneliness among the latter. On the contrary, the study shows that effective communication with parents regarding the illness enhances young people’s sense of support and their ability to cope with parental cancer.

Although the topic of communicating cancer to children has received some attention in the last few decades, Xu et al. (2024) in their recent systematic review affirm that parent–child communication has not been yet studied in depth and more research is needed on communication between parents with cancer and their children. Furthermore, there are additional important aspects that, in our perspective, have been little explored in the literature. First, to our knowledge, only one study conducted in Australia has examined the needs of parents who have to communicate a cancer diagnosis to their minor children (Schiena et al. 2019). This study highlighted parents’ need for professional support, understanding of children and ablility to meet their needs. However, one single study is insufficient to fully address the complexities of this matter, exploring parents’ needs in a specific cultural environment is essential for a patient-centered healthcare system in order to address their specific needs and, consequently, better support parents and their children as well.

Second, no known studies have specifically investigated parental beliefs regarding illness disclosure to their children, despite documented evidence in the literature of a bidirectional relationship between beliefs and communication (Buchanan et al. 1996; Beadle et al. 2004). Understanding parental beliefs is fundamental for promoting effective communication and, consequently, fostering children’s emotional adaptation and well-being (Zhao et al. 2024).

Finally, no known studies have been carried out in the Italian context. Given that the way individuals communicate is deeply rooted in the culture of reference, it is essential to examine both what is communicated and how it is expressed within a specific cultural context (Gordon 1991)

As part of the broader project “Pathways of support for children and adolescents in an onco-haematological context: Support, Research and Education,” funded by the Italian Ministry of Labour and Social Policy, this study aims to explore: (1) the beliefs and strategies parents with cancer use to communicate diagnosis, prognosis and treatment to their minor children and (2) the needs of these parents.

## Methods

### Research strategy

In line with the research aim, this study adopted a qualitative research methodology through a constructivist approach. This approach has been selected as the objective of this study is to achieve an in-depth understanding of how parents experience a specific phenomenon (to be parents with a cancer diagnosis and having minor children); this method is capable of capturing the complexity of subjects’ individual perceptions, feelings and experiences (Braun and Clarke 2021, 2024).

### Participants

The research sample was determined using purposive sampling. Between January and September 2024, all patients with minor children (<18 y.o.) who sought psychological support at either the Clinical Psychology Unit or the Palliative Care, Pain Therapy, and Rehabilitation Unit of the National Cancer Institute of Milan (Fondazione IRCCS Istituto Nazionale dei Tumori) were approached in person and invited to participate in the study. The inclusion criteria were as follows: (1) having a cancer diagnosis or being a partner of a person diagnosed with cancer; (2) having at least a child under 18; (3) willing to participate; and (4) proficient in the Italian language. Approximately, half of the recruited patients were asked to consent to their participation, while the other half were asked to authorize contact with their partner (where applicable). The number of parents to be interviewed was not predetermined; recruitment continued until meaning saturation was achieved. Meaning saturation indicates the gathering of enough facets to sufficiently comprehend information regarding the topic of interest (Sim et al. 2018). Therefore, the analysis was conducted throughout the process of patients’ recruitment.

### Instruments

Each participant took part in a semi-structured interview. Participants were encouraged to recount in detail their experiences, perspectives, and emotions related to the research objective. The interviews were conducted by BS (F, PsyD) and SA (F, PhD), both psychologists and experienced researchers in interviewing. Interviews were conducted face-to-face, by telephone or via the Teams platform.

An interview guide (Appendix 1 – Interview Outline), developed by the research team [SA, BS and CB] and based on the study’s objective, was used to structure the interviews, ensuring that all topics relevant to addressing the research aims were discussed. Additional topics that emerged during the interview, but were not included in the semi-structure interview guide, were taken in consideration as relevant to the aim of the study. The interviews were audio-recorded with participants’ consent and transcribed verbatim. The mean duration of interviews is 36 minutes.

### Analysis

The transcripts were analyzed using Braun and Clarke’s reflexive thematic analysis (RTA) (Braun and Clarke 2019, 2021, 2024). RTA is the method chosen to construct meaning from themes informed by interviews. In line with the chosen approach, no themes were identified a priori, instead a bottom-up approach was used. Aiming to achieve richer interpretations of meaning (Braun and Clarke 2019, 2021), three researchers [SA, BS, ZN] read the transcripts several times simultaneously to gain an overview of the material and the meaning of the content, keeping the research objectives in mind. Quotations useful for answering the research objectives were selected and grouped into codes. A more detailed analysis was then carried out through the researcher’s active role in generating themes and sub-themes and to find links between them. This process was iterative (Braun and Clarke 20021). The transcripts were analyzed using “pen and paper.”

No separate analyses were conducted regarding the role of the parents (mother/father, patient/partner), both because the research objective did not require it and because the parental couple was considered as the unit of analysis (Scabini and Iafrate 2019).

### Ethical issue

The study was conducted following the Helsinki guidelines for research involving human subjects and was approved by the INT Ethics Committee (INT 281–23). All participants signed a written informed consent form for their participation in the study, for their privacy and for the audio recording. The interviews were anonymized to ensure privacy.

## Results

### Participants

Eleven parents were asked to participate, but one declined due to a lack of time and interest in the research. Ten parents participated in the interviews, whose characteristics are shown in Table 1. Meaning saturation was reached at the seventh interview; however, three further interviews were conducted to ensure the identification of new facets relevant to better comprehend the phenomenon. Participants were not patients of the interviewers.

**Table 1.** Participant characteristics

Interview Ref	Sex	Role	Year of Birth	Patient/partner’s diagnose	Clinical phase of care pathway	Relationship Status	Education	No. of children	Children’s age	Children’s sex	Length of the interview
1	F	Patient	1982	Breast carcinoma	In active treatment	Married to the father of her children	PhD	2	3/6	M/M	00:26:48
2	F	Patient	1979	Metastatic breast carcinoma	In active treatment	Divorced from the father of her child and currently in another relationship	Lower secondary school diploma	1	18	M	00:45:43
3	F	Partner	1971	Colon stenosing adenocarcinoma	Under palliative care	Married to the father of her children	Secondary school diploma	3	19/18/16	M/M/F	00:54:56
4	F	Patient	1969	Breast carcinoma	In active treatment	In a relationship with the father of her children	Secondary school diploma	2	18/15	F/F	00:37:34
5	F	Patient	1978	Metastatic breast carcinoma	In active treatment	Married to the father of her child	PhD	1	8	M	00:34:38
6	F	Patient	1976	Intestinal neuroendocrine tumor	In active treatment	Missing	Missing	3	21/17/13	M/M/M	00:32:38
7	M	Partner	1983	Oral cavity squamous cell carcinoma	In follow up	Married to the mother of his children	Secondary School Diploma	2	13/9	M/M	00:34:13
8	F	Patient	1972	Colon stenosing adenocarcinoma with metastasis	Under palliative care	Married to the father of her child	Secondary School Diploma	1	17	M	00:42:02
9	F	Partner	1980	Hypopharyngeal squamous cell carcinoma	Follow up	Married to the father of her children	Master’s Degree	2 (twins)	5/5	M/M	00:35:17
10	M	Partner	1974	Breast carcinoma	Follow up	Married to the mother of her children	Master’s Degree	2	5/3	F/F	00:19:29

### Theme generation

Thematic analysis led to the creation of five themes, listed and defined in Table 2: (1) the challenges parents faced at this sensitive time; (2) the emotions parents experienced; (3) the beliefs that may have influenced how parents communicate the illness to their children; (4) the strategies parents used to communicate the illness to their children; and (5) parents’ perception of their children’s understanding of the illness. Subthemes were also identified for each theme. Sample quotations are presented in Tables 3–6.

**Table 2.** Identified themes, definitions, and sub-themes

Theme	Definition	Sub-themes
The challenges parents faced at this sensitive time	Reports from parents about the challenges they have faced or are facing with their children during their illness and/or treatment.	1. Communicating cancer to their children.
		2. Dealing with the challenges of everyday life while having cancer.
The emotions parents experienced	The emotions parents feel about communicating with their children and/or their role as parents during this delicate time.	1. Concern
		2. Guilt
		3. Loneliness
		4. Fear of a physical and/or emotional breakdown.
The beliefs that may have influenced how parents communicate the illness to their children	Parents’ beliefs about their children’s ability to understand cancer, and how to communicate with them about it.	1. What and how to talk about the disease.
		2. The role of professionals
The strategies parents used to communicate the illness to their children	The strategies parents use when communicating or hiding their illness from their children.	1. Parents who prefer to remain silent.
		2. Parents who prefer an “open” approach.
The parents’ perception of their children’s understanding of their illness	What parents think their children understand about the illness, the treatments and so on.	1. Uncertainty about what children have understood.
		2. Similarities in communication styles between parents and children.

**Table 3.** Examples of quotations of the theme “The challenges parents faced at this sensitive time”

Ref. quotations	Examples of quotations
1	*My daughters were, of course, worried when I was in hospital. But my partner and I always reassured them that I was OK. I never really discussed it with them because I was afraid of making them anxious.* (Patient 4)
2	*The hardest part was telling him that his mom had cancer. Cancer that had spread. It was tough for me to accept, and it was hard to tell my son.* (Patient 2)
3	*My constant fear is that I won’t be able to strike the right balance between transparency and imagination. Failing to manage their reactions is perhaps another fear. I’m afraid of not knowing how to manage their reactions or being unable to predict them correctly. So, when you say something, you never know if it will be good for them or not.* (Patient 1)
4	*At this stage of the illness, I’m afraid I might get a third tumour, perhaps in a different place. I no longer have any certainties; one tumour could be an unfortunate one-off, but two start to be worrying.* (Patient 4)
5	*It wasn’t easy. On top of that, I also had to take care of my dad. He’s not senile, but he’s in his 70s and has health problems, so I had to take care of him too.* (Patient 5)
6	*Last year, I also sought support because my mother had died of cancer. I was about to start chemotherapy and was worried that my son would link his grandmother’s hair loss to her illness and then see me losing my hair. I was afraid he would make the connection and say, “Oh no, Mummy is going to die too.”* (Patient 5)
7	*It’s difficult because children have such specific backgrounds, which makes everything very complicated. Speaking for myself, I am in a difficult situation. I’m divorced. The father is completely absent, even in this situation. Today, I feel guilty about having a difficult separation and about having a debilitating illness like mine.* (Patient 2)
8	*You must consider that I am separated, so I am alone in dealing with these situations. Most of the time, if not always, the children are with me. Their father has not been a source of support, except during the week I was in hospital for an operation. I needed to regain control beyond the illness itself.* (Patient 6)
9	*Losing my job affected me the most because I had worked at the same small company for 26 years. We all knew what everyone else was having for breakfast, so to speak. I thought we were friends, but I realised that wasn’t the case. That disappointed me in many ways. Even from a human point of view.* (Patient 8)
10	*I try to be a mum like I always have been. Controlling everything. But there’s an illness in the middle of it all. I’ve always tried not to think too much about my illness and what the future might hold. I try not to think about my illness too much and just get on with my life. Of course, there are days when I’m really tired, physically and mentally. I try not to think about it too much though, and I do exactly what I would have done if it weren’t there. It’s not easy, is it?.* (Patient 2)
11	*Luckily, he’s only eight, and I’m glad I don’t have to do certain things anymore, like hold him in my arms.* (Patient 5)
12	*I miss being able to support him in lots of ways, like going with him to school or on trips. I can’t go with him in certain situations, either physically or in my free time, because I don’t have the stamina. My health doesn’t allow me to.* (Patient 8)
13	*If you look at the negative side, so to speak, there’s the absence of the father, who perhaps can’t wrestle with his children as he used to. To sum it up, we’re a bit worried that these things might affect their growth later on. But, I totally get that you do your best and keep going like this.* (Patient 9)
14	*The things I used to do with him, like riding bikes, are pretty much a thing of the past now. I would have liked to go skiing with him and do lots of other things together. I do feel a bit bad about it sometimes. I do them anyway, and if the doctors catch sight of me, they tell me off. But I know my limits, and I try to do a little bit here and there.* (Patient 5)
15	*I’ll be honest with you, there are days when I’m really tired, but more mentally than physically, and he [my son] doesn’t help me, even though he doesn’t mean to. He has to find his own path and make his own way, so it’s a daily struggle. He just keeps on doing his thing, and of course, he has his needs. Then there’s this girl, going here, going there. It’s his life. You’ve got to keep going, because otherwise it’s a disaster. It’s a delicate life. It’s a job where, as I always say, you can’t afford to be away from the office. You can’t afford not to be ready to control him. I’ve got my eye on him all the time. I’m a mum.* (Patient 2)

**Table 4.** Examples of quotations of the theme “The emotions parents experienced”

Ref. quotations	Examples of quotations
16	*As a mum, you have to be there all the time, for everything. It’s not easy, because it’s a very special age [..] It’s tough, I’ll admit, because I’ve also had moments where I felt discouraged.. afraid, really afraid. I’d lock myself in the bathroom with the tap running so that no one could see me crying or being afraid. My son never saw me on my knees. He was there for me when I needed a helping hand, but that was just when I had no energy. Even with the chemo, I was never really sick: I slept, so I just needed to recover my strength. Even when I was feeling down, I always tried not to let him see. I put up with everything, and it’s not easy. It’s a situation that tests you every day. And when you’ve got a 17-year-old son, it’s already tough. It’s not easy to deal with the difficulties and everything else.* (Patient 2)
17	*I think the hardest part is breaking the news to them. In our case, my husband wanted to tell them at the dinner table. It was a bit of a tough one because he couldn’t speak. I think it was the first time they’d ever seen him cry, because my husband’s mother had had cancer, and he didn’t want them to go through the same thing, so he started from there. It was a tragedy because, in any case, there were.. they [my children] were rightly moved, so it was very touching, very nice, in a way, very heartfelt by everyone, and I don’t think it was difficult because, in any case, it was something quite.. well, these things happen. The tricky bit is then making it seem natural to them, I mean natural: it’s life! I mean, it can happen, and that’s why you have to move on, so that’s the hard part.* (Patient 3)
18	*When we got home and had to tell the kids that their dad had to have an operation and that he would no longer breathe through his nose and mouth, but through a hole in his neck, it wasn’t easy. It was a bit of a hassle, to be honest.* (Patient 9)
19	*My main concern is always to be able to stay close to him and see him grow up [my son]. That’s my main concern. You always hope he’ll stay there and not go into decline. Then, little by little, the concerns are always about how to tell him things, how to deal with him.* (Patient 5)
20	*As a mum of two girls, I’m worried about their future. I had two breast tumours myself, so I think they should be included in a monitoring programme. But it’s usually up to the parents to take the lead. I haven’t really been given any specific advice on how to proceed for their future, or any advice on what to do for prevention, when to start, and all that.* (Patient 4)
21	*We never said, ‘Look, mummy might one day.. [die] because I don’t want to burden him with my worries. Those are my concerns and I’m not going to pass them on to you.* (Patient 5)
22	*The most important thing is that, sorry for the expression, in this “crappy” situation, my son lives in peace. That’s what I’m worried about. That he tries to do his best, and keeps on doing that. That’s why I carry on with my normal life, I suppose. I go to work. I try to do the things that all mums do, as well as I can.* (Patient 5)
23	*I know that at some point, you just get on with it and figure it out. I’d like him to do more things independently, but there’s just no way. I’m already feeling a bit guilty about this, because I haven’t been able to teach him certain things from the very start.* (Patient 8)
24	*The meetings with Dr [name of psychologist] are also fairly recent. I realised how much the information I received in November and December had thrown everything into disarray. I had to stop and think, “OK, enough is enough. I need to take a step back and reset, because otherwise it’s just not working. You can’t afford to keep going like this.” You must consider that I am separated, so I am alone in dealing with these situations. Most of the time, if not always, the children are with me. Their father has not been a source of support, except during the week I was in hospital for an operation. I needed to regain control beyond the illness itself. […] From January onwards, the things I did […] meant I was like, “You can’t afford it, period.”* (Patient 6)
25	*I’d say the hardest part is trying not to lose my emotions. I always try to push these thoughts away, but I tell myself, “He’s eight years old today, who knows how many birthdays I’ll be able to celebrate with Tommaso.” I always find myself asking these questions when I’m with him, so if I start to feel sad or worried, I try to push them away. It’s just that worry you get as a mum, you know, of wondering, ‘How long will I be there for him?* (Patient 5)

**Table 5.** Examples of quotations of the theme “The beliefs that may have influenced how parents communicate the illness to their children”

Ref. quotations	Examples of quotations
26	*I haven’t really talked to my kids much about my illness, just the basics, even though they knew about my hospitalisation and the operation. They also found out that I was at my mum’s for a bit, so the youngest was with me, while the other two basically lived between their grandma’s and our house. So they definitely experienced the illness, but I didn’t talk about it much, just the necessary info.* (Patient 6)
27	*One of my two daughters, the older one, tends to get really anxious, to the point of illness, and maybe partly because she’s seen me going in and out of hospitals, going to the doctor all the time, every day’s a different challenge, so I see that she’s already becoming a bit hypochondriacal.* (Patient 4)
28	*It’s clear that when they were younger, they didn’t have this ability, but now that they’re 13 and 9, they can recognise when their mum’s feeling down and not push her.* (Patient 7)
29	*Kids often want answers as well. When you talk to them about something, they don’t understand that there are expectations and uncertainties, so they get really agitated, maybe even more than adults do. Basically, they have trouble sleeping, and that can show itself in other ways. You also have to watch out for them getting down about things that might turn out well in the end. I think it’s important to be a bit careful about expectations and not always stress them out. I’ll let you know when this happens.* (Patient 3)
30	*We haven’t really gone into it in any great detail; [communication] we keep it pretty simple for the kids because they just aren’t able to understand certain emotions. It’s important to keep communication fairly straightforward at their age without going into things that are too difficult.* (Patient 7)
31	*My son never saw me on my knees. He was there for me when I needed a helping hand, but that was just when I had no energy. Even with the chemo, I was never really sick: I slept, so I just needed to recover my strength. Even when I was feeling down, I always tried not to let him see.* (Patient 2)
32	*I want my daughters to be happy and carefree.* (Patient 4)
33	*I always question myself a lot, so I’m always afraid that maybe my husband’s absence, even now that he’s at home.. I’m very happy that he’s at home because he’s more present. If you look at the negative side, so to speak, there’s the absence of the father, who perhaps can’t wrestle with his children as he used to. To sum it up, we’re a bit worried that these things might affect their growth later on.* (Patient 9)
34	*The tricky bit is then making it seem natural to them, I mean natural: it’s life! I mean, it can happen, and that’s why you have to move on, so that’s the hard part.* (Patient 3)
35	*We also did video calls with the hospital when my husband had complications with the chemo, he got pneumonia and was admitted. We were already doing video calls while he was having chemo. So we showed them what the hospital room was like. We always told them the truth, basically to prevent them from feeling guilty and imagining things.* (Patient 9)
36	*His dad was really supportive, and we tried to make him see that we need to be patient, that this is only temporary, that the doctors are good and that there’s always good and bad in life. We try to talk to him.* (Patient 1)
37	*I’m not sure if that’s correct, but I told them that “Dad had to stay in hospital because they cut out the disease, the tumour he had.” So, unfortunately, they also removed some things that we have in our bodies that we need to talk. So basically, I explained it to them like this: “If you read his lips – and I should mention that they’re better than me at reading lips – they understand, but at the moment he doesn’t have a voice. So later on, when he gets better, Dad will be able to use tools that will allow him to speak.” “He won’t have the same voice as before, but his voice will be a little different, maybe a bit like a robot, but he’ll still be your dad.” That was the last thing I said. First off, I mentioned that my husband had radio-chemotherapy too, which made the whole thing pretty long. He lost his hair and a lot of weight while he was having chemo. I tried to explain to my youngest child that these changes happened because of his dad’s treatment. I told him that he was losing his hair, but it was helping him get better.* (Patient 9)
38	*I don’t think external factors, like advice on having or raising children, can be the right thing. I’d have to see a shrink for two years, talking about my kids’ lives and everything that goes on in their heads and what they’re like, just to get help from a third party on how to behave with each of them. If I ever talk about it with outsiders, such as my mother, who’s always around the house, or with people who know the family and live in the family circle, then we can discuss it. This can also be useful for us as parents. But I think the main help from outside should go to the parents, the patient and their partner. And then for the kids, where, as I was saying, I think it helps everyone, regardless. I’m all for it, as I said before, because children don’t always express themselves and not all families are the same.* (Patient 3)
39	*I think parents and children should be able to support each other in whatever way the parents decide. I mean, the choices we’ve made in our circle are personal and subjective, and if someone else had dictated them, they wouldn’t be right for everyone. Because family dynamics are very different in each family, and depending on how fragile people are, parents have to make choices, in my opinion. So, depending on the period, we made this choice based not only on age, because anyway.. but also on how fragile the person was.* (Patient 3)
40	*It was something that had to be done, and I did it my way. I just followed my gut.* (Patient 6)
41	*It’s like education, or like.. I’m not sure. I just go with my gut. I always speak to him from the heart.* (Patient 2)
42	*I think it comes naturally in a family with two parents, but, as I said before, every family is unique and every family recognises the ways and weaknesses of their children. So, every family needs different support.* (Patient 3)
43	*I’m the only one who can tell my son what I want to say. I can see the logic in saying it that way, but I need to experience it first-hand, you know, to get that feeling of “I’d rather talk about this than that,” or to understand that I said that, I saw that reaction, so it’s probably better that he doesn’t know about this other thing.* (Patient 8)
44	*So I took steps to make sure I had a plan for how to proceed. People are often afraid of making mistakes, so they ask what the right way is. Then, if they see that the approach they’ve taken is working, they don’t ask any more questions, they see that it’s fine. If I’d seen that things could have gone differently, I definitely would’ve asked for help and support.* (Patient 5)

**Table 6.** Examples of quotations of the theme “The strategies parents used to for communicating the illness to their children”

Ref. quotations	Examples of quotations
45	*I didn’t go into detail about what a tumour is because I didn’t want to make them anxious or worried.* (Patient 4)
46	*I think it was more about not wanting to burden them with more than they needed to know. There was nothing they could do about it anyway, and it would have been better for them not to worry about it. It’s not fair that they should suffer more than they have to. The plan was to avoid panicking in a situation where I wasn’t actually in any danger.* (Patient 6)
47	*I’m talking about my eldest daughter, who is 5 years old. She saw some little cuts caused by the tubes they use for therapy. At first, maybe just as a joke, her mum said, “It was Lola,” our dog, “who scratched me.”* (Patient 10)
48	*They don’t want to know much. They ask, “How are you?” and I say, “Fine.” Or I’ll just say, “I’ve got a headache, I’m tired.” They ask me, and I rarely hear them ask their dad, “How are you?” They obviously understand that I have some health problems, I do indeed.* (Patient 4)
49	*I’ve always tried not to think too much about my illness and what the future might hold.* (Patient 2)
50	*I didn’t go into detail about what it could mean for the future. I always reassured them. I said, “I’ll have the surgery now and we’ll sort it out.”* (Patient 4)
51	*The disease is treated in a way that fits in with everyday life. In a really calm way. [..] I don’t think about my illness all that much, unless I faint. I just get on with my normal life. Obviously, there are days when I’m really tired, physically and mentally. I try not to think about it too much and just do what I would have done if it weren’t there.* (Patient 2)
52	*I try to do the things that all mums do, as much as I can. Obviously, if I’m feeling knackered, I can’t do it – I’ll take a break, because I’m not Wonder Woman. But I try to keep things as normal as possible.* (Patient 5)
53	*For now, I’m not in any danger. I’m not like someone with advanced cancer who needs to prepare their daughters to live without them. We’re not quite there yet, so there’s no point in stressing them.* (Patient 4)
54	*Sometimes, my main concern is what they’ll do when I’m not around anymore [my son and his father]. What about the everyday things – will they be able to manage those? I realise it’ll be tough at first, but they’ll get the hang of it and be able to continue without.. [me]? I’m not sure. I’m not worried about not being there for myself, I’m more concerned that they won’t be able to manage on their own. I know that at some point you just get on with it and learn as you go. I always try to.. I’d like them to do more things independently, but there’s just no way. I’m already feeling a bit guilty about this, because I haven’t been able to teach them certain things. I don’t know how they’ll manage to keep going without getting overwhelmed by things. Sometimes more than submerged, “completely swept away.”* (Patient 8)
55	*But with them, I’ve always tried to avoid going into too much detail.* (Patient 4)
56	*Right from the start, when I had to have a colonoscopy, I explained to him that the test had come back positive, even though I had gone in for the colonoscopy feeling quite calm because there are a lot of false positives. But then we were like, “Look, it’s gonna be a long road ahead,” because the news just kept getting worse and worse. At a certain point, I was like, “Wait, let’s stop here, because I can see he’s already too….”* (Patient 8)
57	*I didn’t think I needed any support to talk to my kids about certain things, because I saw it as: ‘Guys, we’ve got to do this, no ifs, ands, or buts. I’m telling you, I’m doing this, I’m doing that, I’ve done this, now let’s do something else. That’s it. […] I was pretty concise in my communication, just the essential info. “I’ve got a check-up booked for a certain day. I went for my check-up and everything was fine.”* (Patient 6)
58	*I said, “Mum’s getting treatment. She’ll need treatment for a good while. As you can see, I’m doing well. So, we’ll just keep going with that.” The only thing that mattered to him was seeing that I was OK. Everything else was secondary, wasn’t it?.* (Patient 2)
59	*At home, we don’t use the word “cancer,” OK? Even though “tumour” and “cancer” mean the same thing. I’ve got a bit more of a cancer than a tumour, because having stage IV metastatic cancer was tough, but we try to keep things as light as possible, because it’s already pretty heavy.* (Patient 2)
60	*I don’t think we used the term “tumour” with the 12-year-old.* (Patient 6)
61	*My youngest son knows about the tumour and the primitive mass, but we haven’t talked about metastasis because of his age. We’ve been way more honest with the older kids because they’re older, and we’ve always seen it in a very positive light.* (Patient 3)
62	*They knew about the cyst and that Dad needed to get it checked out. He also had one on his head that was definitely an incised cyst, which had gone away, so we just hid the fact that it was probably a tumour too, until it happened and things became clearer.* (Patient 3)
63	*They know about my breast problems, lumps and surgery. They’ve never asked me what it was, though. I didn’t go into detail about what a tumour is because I didn’t want to worry them.* (Patient 4)
64	*I was told on Friday that I’ve got to have radiotherapy on my head. They’re going to put a mask on me until there are any changes. I don’t think I’ll have to take any more stuff off. I’m not saying anything to them just yet, there’s no need to tell them. Everything’s still on as normal, and Mum’s having her treatments.* (Patient 5)
65	*I’ve always been open with him though. Not in a brutal way, though. Even now, he knows that I need to have surgery. He knows the whole process and what will happen, bit by bit. But he knows everything.* (Patient 2)
66	*My wife is also having surgery next Monday. We’ve tried to deal with it together with our daughters. Last night, we also let the kids know that their mum is going to have a bit of a procedure next week. They knew their mum would be away for a few days. We’ve done our best to weigh everything up, as much as we can, obviously in a new situation.* (Patient 10)
67	*I’d say that, physically speaking, I was at my weakest between January and March last year. I was in a lot of pain and couldn’t walk very well. I mean, I needed a hand to get up and climb the stairs. I couldn’t manage these things on my own anymore. I think that was the worst time for them too, in the sense that they could see that I was physically unable to do things normally, that I needed help even for the most trivial things, like getting up from a chair. Once I was standing, I could walk, but once I sat down on the sofa, I needed to hold on to something or have someone help me up. That was the worst moment, because whatever I said, there was one thing I couldn’t say. I was obviously unwell, so knowing the diagnosis, it was the worst moment for me, because I had no strength, and for them, because they had to face the situation and understand that it had reached a certain level.* (Patient 8)
68	*They’ve definitely picked up on something, but we told them last night that she’d be going straight into hospital. They seem to have understood that in four days’ time their mum won’t be at home and that she’ll have tubes in her for the following days, so she needs to rest.* (Patient 10)
69	*When he was scheduled for surgery, they did a biopsy and it turned out to be sarcoma, so we waited a while. We took a couple of weeks to process the news, and once we’d spoken to the oncologist about the type of treatment, we talked to the kids right away and told them, “This is what happened.”* (Patient 3)
70	*I had a chat with him. I sometimes did the right thing. My son would also give me a hand now and again. When I lost all my hair and had a wig, and then I had to tell him because I couldn’t wear the wig all the time, he was the one who said to me, “Mum, don’t worry, don’t care, take it off!” He was the one who gave me the strength to face so many things along the way. We help each other out.* (Patient 2)
71	*Nah, I just put it to low with them. I always minimised everything, I mean, I was always very calm. So they were definitely worried when I was in hospital. But I always told them, and my partner too, that everything was fine. I never really discussed it with them.* (Patient 4)
72	*Today, I explained to them that I’m dealing with an illness myself, and that “Mum has discovered she has a completely curable illness,” so she’s currently undergoing a series of treatments to make her stronger. That’s what they know, that’s what they’re experiencing.* (Patient 1)
73	*She doesn’t have a tongue, so there are times when we all joke about the way she eats, because there are certain foods that she finds more difficult to swallow. There’s always the question of what food she can eat and what substances she can have, so it’s always quite an open topic.* (Patient 7)
74	*I told him because when I’m in the shower with him, he can clearly see that one of my breasts is different from the other. I told him about the bad cells that needed to be taken out and that the doctors had given me a fake breast.* (Patient 5)
75	*Telling the truth “in a childlike way” to my children. I found myself in the vortex of illness and so I did the same thing for myself.* (Patient 9)
76	*The first part was that, because my husband also had radio chemotherapy, it was a very long process. While he was having chemo, he lost his hair and a lot of weight. As my husband went through these changes, I tried to explain to our children, together with him, that their dad’s treatment was the reason for these changes. I told them that he was losing his hair, but it was helping him get better.* (Patient 9)
77	*“Look, Tommy, my hair’s growing, even now with the new meds I’ve started,” I told him. “Well, let’s see what mum says, because I might lose my hair. It’s 50 − 50, let’s see if I lose it, let’s see if I don’t lose it.”* (Patient 5)
78	*So the only thing I can think of is that maybe talking to them is the only way, but always, at any time, maybe in a slightly different or more thoughtful way, trying to understand what is going on. Basically, just being really on the ball with these things. If you want to pay attention to these things, you have to be clear-headed, there’s no way around it!* (Patient 3)
79	*During this whole process, the kids weren’t considered because the wait between exams wasn’t weekly, but longer. So, there was a pretty long wait. For me, rather than having precise information, I prefer uncertainty.* (Patient 3)
80	*I didn’t go into any specifics. I just gave her the bare minimum so she wouldn’t get more stressed. Then I don’t know if I’m saying the right or wrong thing, or if I’m just making things more complicated. I don’t know what the point is, though, when everything feels uncertain. What’s the point of making teenage girls worry when they just want to enjoy being a teenager? If I don’t know how it’ll turn out, if it’ll turn out well, maybe it’ll turn out badly, I don’t think there’s any point in me saying too much.* (Patient 4)
81	*Yes, because I always tried to say that I believed in the doctors, that I would undergo the treatments even if I felt bad, I would undergo them, saying: “They are necessary to make me feel better” and I tried to instil this attitude in them too, especially in my son. “Look, even if you see me like this, it’s because this is what makes me feel like this, but it’s necessary to make me get better.”* (Patient 8)
82	*A patient is a patient, and a child is just another patient, right? So you shouldn’t put them in a position where they feel distressed by the illness. Try to see it as positively as you can. I think that in my case, whoever’s in charge of managing everything should be kept a bit more in the loop, just to avoid any nasty surprises.* (Patient 3)
83	*Things are pretty calm at the moment. Normal life has resumed peacefully. Now life is going normally, more than ten years have passed since the illness [..] Today, for today.. The good thing is that [name of partner] has always been really open with the kids about what happened, so I’d say the best thing we’ve done with the children is to keep talking about the illness, because even now they can talk about it openly. I’d say the whole thing’s going on inside [partner’s name] body, so it’s down to her. She’s a pretty direct person, after all. It makes no sense trying to keep them out of it or pretending [..].* (Patient 7)
84	*My wife said, “Yes, let’s tell him.” A couple of months passed after we first discussed it, but my wife’s words are still stuck in my mind: “It’ll be one less thing to worry about once we’ve told him,” so it’s a positive thing.* (Patient 10)
85	*It’s exactly what happened with my parents. Maybe they unconsciously said something to him that I hadn’t said, and I turned into a hyena. I’m the one who talks to him, so you’ve got to support me. But the way I say things, the way I.. handle them, that’s up to me.* (Patient 2)

**1) The challenges parents faced at this sensitive time**

Parents report facing many challenges in their relationships with their children during the illness. Two issues in particular emerge: communicating cancer to their children and dealing with the challenges of everyday life while having cancer (for quotations examples please see Table 3).

**Communicating cancer to their children.** Everything related to this topic was generally very difficult. Particularly critical moments included the diagnosis, worsening health, hospitalization (ref 1) and appearance of metastases (ref 2) (for the latter see also the topic “Strategies for communicating the illness to children”). However, uncertainty about what children have understood could also be stressful (see the topic “Perception of children’s understanding”), because parents did not know “how to act.” It was particularly challenging to address children’s concerns about their parents’ health (ref 3), which is why parents often chose to postpone the communication of their diagnosis.

**Dealing with the challenges of everyday life while having cancer**. Everyday life presents personal, relationship and parenting challenges for everyone. For those with cancer, these can become real obstacles due to the physical and emotional burden of treatments and frequent check-ups, as well as the fear of recurrence (ref 4). The interviewed parents mentioned some of the many challenges they faced, such as caring for or mourning the loss of elderly parents (refs 5–6), dealing with arguments, separations or divorces with their partner (refs 7–8), and managing work issues (ref 9). All of these challenges were associated with the complexity of never being able and never wanting to stop “being” a parent (ref 10), i.e. remaining focused on the needs of their children in both a psychological (ensuring they live a “normal” life, not burdening them with the disease and finding the energy for carefree activities) and physical (holding them, taking them to school and recreational activities) (refs 11–14). For all these reasons, many of the quotations collected point to a fear of “giving in” (ref 15) (see also the “Emotions” theme).

**2) The emotions parents experienced**

This theme only covers emotions related to the parent-child relationship, not those related to the illness itself, however present and impacting QoL of families as a whole. Parents experienced a wide range of emotions concerning their children, and in most cases, these emotions were intertwined. Here, however, emotions will be distinguished solely for reporting purposes. For quotations examples please see Table 4.

**Figure 1.**
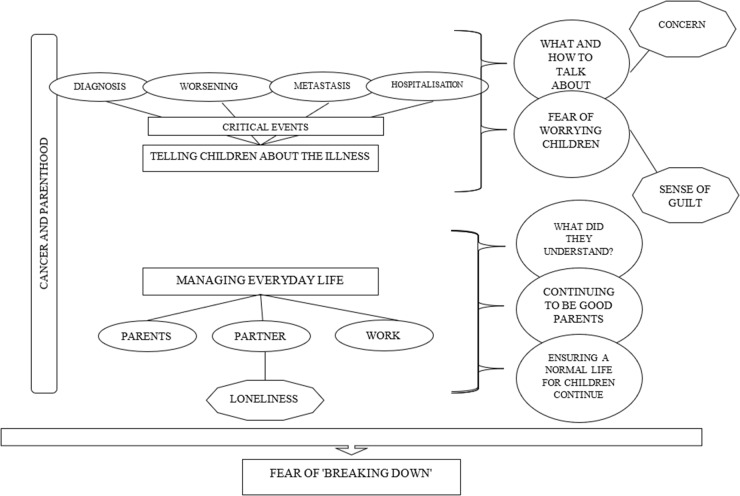
A graphic summary showing the conceptual connections between the themes “The Challenges parents faced at this sensitive time” and “The Emotions parents experienced”.

**Concern.** There were numerous concerns, many of which were related to appearing “sick” or “weak” in the eyes of children (ref 16), especially when it was not possible to allow them to live a “normal” life, i.e. the everyday life they had before the illness. For the interviewed parents, being a “good parent” appeared to require emotional and physical “strength.” Showing themselves in tears or bald seemed to transmit a “negative message” to one’s children.

Communicating the illness to children in an appropriate manner was also a concern, particularly during hospitalization or when the condition worsened (refs 17–18). The underlying premise is that inadequate communication management may negatively impact childen’s well-being (See topic: “The beliefs that may have influenced how parents communicate the illness to their children”).

The worry about not seeing their children grow up was sometimes verbalized, however, it was more often kept silent (ref 19). Researchers perceived that certain parents were afraid to even discuss this option. Hence, to respect this belief, this matter was not further investigated.

Another concern was related to passing on the disease to their children and not being able to provide them with the best diagnostic and therapeutic tools to prevent or support this eventuality (ref 20). This concern arises from the perception that the National Health Service frequently fails to address patients’ needs promptly and effectively.

Finally, a further concern, presented only among the mothers in this study, was related to not being able to adequately “equip” their children for life in case they die. This worry may also reflect emotions of guilt due to the perception of “not having done enough” for one’s own children (also see sub-theme “Guilt”).

**Guilt.** Often linked to concern, guilt emerged. It was associated with the worry of “overburdening” children with a burden that a parent would like to spare them (ref 21), as well as with not allowing children to live a carefree life (ref 22). Some mothers, who knew their illness was worsening, also felt guilty for not teaching their children to be more independent and, therefore, to “fend for themselves” when they will pass away (ref 23).

**Loneliness.** Many of the comments expressed a sense of loneliness, particularly among single parents (ref 24) and those without help from grandparents. These individuals had to manage the illness while raising children and coping with the daily grind (see also the theme “challenges”).

**Fear of a physical and/or emotional breakdown.** The numerous daily challenges and struggles, both psychological (the whirlwind of emotions) and physical (frequent exams and check-ups, the side effects of treatment, etc.) led parents to fear that they would not be able to cope (ref 25). Added to this was the thought that they couldn’t afford to give up both because their young children needed them, and because they felt children should have been shielded from witnessing their parents in pain (see also the theme “Beliefs”). Knowing that their children rely on them for survival reasons function both as a source of strength and as an amplifier of the emotional burden and duty carried by parents. Figure 1 shows a summary of the “The Challenges parents faced at this sensitive time” and “The Emotions parents experienced” themes, along with the conceptual connections between them.

**3) The beliefs that may have influenced how parents communicate the illness to their children**

This theme presents parents’ beliefs about how to communicate to their children about cancer. Beliefs can be defined as convictions about the world and how it works. For quotations examples please see Table 5.

**What and how to talk about the disease.** The first sub-theme concerns the amount of information and terminology to use when talking about the illness. At opposite ends of a continuum, two extremes were identified: those who believed it was best to “say nothing” to their children about the disease, and those who believed it was important to establish an open dialogue. The former was based on specific beliefs held by parents. One belief was that children are happier if they are unaware of their parents’ illness (ref 26). In this regard, it is interesting to note the account of a mother whose daughter became a hypochondriac after seeing her go in and out of hospital several times (ref 27). Another belief was that children are less able to understand life events (and therefore also their parents’ health) (ref 28), and less able to understand and express certain emotions (refs 29–30). Parents who fell into this category emphasized that their children should not have seen them suffering (ref 31). On the contrary, they argued that it was important for children to always see their parents in good health because the parents’ illness should not have changed the normal course of children’s lives (ref 32). For example, a parent argued that if the father did not behave as he did before the illness, the children could have developed problems in the future (ref 33). In one statement, a parent said that although he understood illness was a natural part of life, he was still afraid of not being able to communicate this effectively to his child (ref 34).

At the other extreme there were parents who believed that maintaining an open communication with their children was important, even when they had to deliver difficult news (ref 35). These parents acted on the belief that this type of communication helps their children better understand and adapt to the disease itself (refs 36–37).

**The role of professionals.** A second sub-theme concerns beliefs about the role of professionals in supporting parents when communicating the illness to their children. This sub-theme also revealed a continuum, with parents who believed that professional support (e.g. from a psychologist) is useful in determining “the right way and time” to inform their children of the illness at one end of the continuum, and parents who believed that only they can determine “the right way and time” and that professional support is, therefore, unnecessary. One end of the continuum comprises comments from parents who sought help from a psychologist and were satisfied with the results, despite some initial scepticism about the value of this support (ref 38). At the other end of the continuum were parents who believed that they alone could find the “right key” to communicate the illness to their children because they know their children’s characteristics, and therefore know when to break the news and what terminology to use (ref 39). These parents emphasized the importance of instinct, intuition and emotion in communicating with their children (refs 40–41). They believed that professionals’ suggestions are too “standardized” to be useful (refs 42–43). They only considered support from a professional in case they would feel that something was “wrong” with their children (ref 44).

**4) The strategies parents used to communicate the illness to their children**

This topic covers the strategies parents used to talk to their children about the disease.

The interviewed parents questioned whether their chosen approach was appropriate. Parents feared that an incorrect communication may cause anxiety in their children (ref 45). They also questioned what information their children needed to know and what should be disclosed (ref 46). Even those who have suggested a light-hearted approach wondered how far they could go, given the serious nature of the subject. Interestingly, some parents focused heavily on what their children needed or wanted to know about cancer, while placing less emphasis on explaining how they felt physically and emotionally. For quotations examples please see Table 6.

**Parents who prefer to remain silent.** In line with the “Beliefs” theme, parents who did not feel able to communicate openly with their children about the disease used communication strategies such as minimizing its impact on their lives (refs 49–50), normalization (refs 51–52), avoiding the concept of terminal illness (ref 53) and reassuring children until they must prepare them for their death (ref 54). Parents emphasized that they have communicated “only what was strictly necessary,” believing that it was better to provide little information (ref 55) for fear that providing more details may cause anxiety in their children (ref 56). Communication was often limited to “practical” aspects (e.g. undergoing treatment), presented as a “shopping list,” where parents were careful not to reveal any fears or concerns (refs 57–58). They therefore avoided using terms such as “cancer” (ref 59), “tumour” (ref 60) and “metastasis” (ref 61), preferring the more reassuring terms such as “cyst” (ref 62) and “lump” (ref 63). Communication often took place shortly before an important event, such as surgery (refs 64–66), or when there was a significant change in the parents’ state of health (refs 67–69).

Some parents said they “know what their children know” about their illness, but preferred to avoid talking about it until their children took “the first step” (see also the topic “Perception of children’s understanding”). A discrepancy could sometimes be perceived between what parents said to their children and what they communicated through their behavior, even if they may not be fully aware of it (ref 71).

**Parents who prefer an “open” approach.** Conversely, parents who opt to discuss the illness with their children emphasized the significance of “constantly maintaining an open mind” regarding the topic. Rather than waiting for their children to bring the subject up, they introduced it themselves (ref 72). A fine illustration of this was a family where the mother, who had an operation for tongue cancer, found it hard to consume certain solid foods. This has turned into a source of amusement and humor during mealtimes (ref 73). This family has found a playful way to relieve the stress of a difficult situation.

For these parents, it was crucial to select appropriate terminology when communicating with their children, ensuring it was age-appropriate and aligned with their level of understanding (ref 74–75). As well as using simple, understandable language, creative methods based on images or simple metaphors could be employed, even with young children. The following patients’ quotations illustrate this: *“He knows that Mummy has black ugly cells inside her body that need to be made colourful again. So she goes to hospital for treatment to make them colourful again. We explained to him that the doctors are working hard to make everything colourful again”* (Patient 5). *“Then I may have exaggerated a little by telling them that Mummy is getting stronger, like a superhero, thanks to this treatment. But I think they’re taking it all in good humour now”* (Patient 1).

Some parents expressed a desire to prepare their children for the physical changes that treatment may have brought. They did this by explaining the possible scenarios that may have occurred (ref 76–77).

However, these parents needed to accepted all aspects of the illness, such as communication of the diagnosis, surgery and treatment, before they felt ready to tell their children (ref 78). By “feeling ready,” parents meant that they had come to terms with the diagnosis or deterioration of their health, and had a clear understanding of the potential outcomes (ref 79–80). This was an essential aspect, as it was based on the idea that they must be calm in the first place to communicate calmly and effectively. Parents tried to tell their kids about the illness whereas giving them hope for the future while they were getting treatment (ref 81).

In this regard, it is interesting to note a parent’s statement that ‘this must be the “correct” communication strategy because it is the same one used by doctors (ref 82).

Another practice observed in the interviews was that communicating the illness to children was mostly delegated to the sick parent, even in the presence of the other parent (refs 83–84), as if it were their task or responsibility. One parent reported that she was solely responsible for providing information to his child and did not accept anything his parents said about his illness, even unintentionally (ref 85).

**Figure 2.**
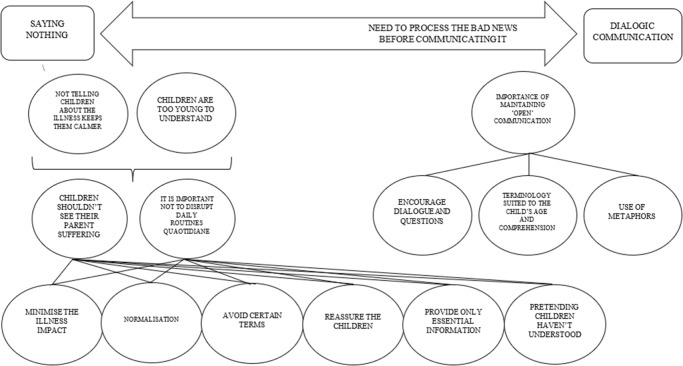
A graphic summary showing the themes ‘The beliefs that may have influenced how parents communicate the illness to their children’ and ‘The strategies parents use to communicate the illness to their children’ and the conceptual connections between them.’

Figure 2 summarizes some results from the themes ‘The beliefs that may have influenced how parents communicate the illness to their children’ and “The strategies parents used to communicate the illness to their children” and the conceptual connections between them.

**5) Parents’ perception of their children’s understanding of the illness**

This topic covers how parents perceive their children’s understanding of the illness (for quotations examples, please see Table 7).

**Table 7.** Examples of quotations of the theme “Parents’ perception of their children’s understanding of the illness”

Ref. quotations	Examples of quotations
86	*The teacher told me she was moved and said: ‘It was a child who told me, “No, if your mum is ill, you must go immediately, you mustn’t stay here, you must go to your mum right away.”* (Patient 5)
87	*Every now and then one of my sons, especially my 6-year-old, makes comments because he sees me in bed “exhausted” during chemotherapy, and he has made a couple of remarks such as, “Come on, Mum, you’re always in bed, you’re always tired,” so I guess that’s his way of expressing discomfort or discontent.* (Patient 1)
88	*Yeah, maybe he wants to know because he’s always worried about side effects and the different treatments. “But what does this do to you?” For example, when I have CT scans, radio CT scans and MRIs, I have them every 2 − 3 months and he keeps saying, “But look, there’s radiation, that’s what makes you nervous,” and I say, “Don’t worry, there are other things.. there’s everything else I take, who knows what that does to me..” [laughs]. If they’re needed, they’re done. Well, he’s got more immediate things on his mind, that’s for sure!* (Patient 8)
89	*So, there are things that come out every day, like she’s more tired than usual or her legs hurt sometimes. So there you go, because it’s just become part of everyday life, even the kids are starting to pick up on when she’s a bit low on energy and all that. They’re pretty good at working together and they’re pretty quiet, so they don’t bother you more than they should.* (Patient 7)
90	*The kids were like, “Okay, so mummy, explain it to me properly,” especially Davide, “explain it to me properly, he’ll have a hole there, so how will he breathe?” I said, “It’s all automatic, isn’t it?” Basically, we explained everything to him, even what he would have to do. Also because they see it. Kids go into the bathroom when their dad is having a shower, so they see it. We live our daily lives with our children and that’s just the way we are.* (Patient 9)
91	*They clearly know us, they can see that we’re nervous and stressed. Maybe the things they didn’t say say it all. The anxious vibes and worries. It’s clear that the relationship has always been good, within the limits of what’s possible.* (Patient 10)
92	*Where do you sense discontent? They’ve been saying that I get fed up and that I don’t see myself as “top notch.” Or, for example, the fact that I wear a wig. I don’t wear a wig at home, I wear a hat. They’ve never seen me without hair, by choice, because I had my hair cut before chemo to match the wig. They know that mum wears a hat because she’s very cold at this time of year. I’m not sure if they’ve just not noticed or if they’re pretending, but this is something I didn’t want to share. Whether you think it’s right or wrong, that’s their call. So, when you’re at home, do you wear a wig? I usually wear a wig or a hat. I’ve got two hats, and they know that Mummy wears a hat even when she’s asleep. They haven’t shown any curiosity or interest in this. It’s become almost normal, because I’m cold.. because that’s just how it is.* (Patient 1)
93	*No, he gets it all. Sometimes it seems like he pretends not to know anything, which is probably normal for his age. But he gets it all. Yesterday I found out that a good friend of mine is going through the same thing, and her daughter has become a good friend of my son. So today they started talking at school. And she was like, “My mum’s not really into the whole therapy thing.” She had surgery, where they took out a quadrant, and then they said she should go for standard therapy. She doesn’t want to do it because she’s afraid. And my son just explained this to his friend; He told me just two seconds before she called me. He said he’s trying to convince her because it’s important to do the therapy. Because my mum did it, she’s doing it. So she knows everything perfectly well. He does sometimes just ignore the problem, though. I think it’s just a form of protection. It’s a protection that we all have and put up.* (Patient 2)
94	*So, over time, it’s possible that they’ve definitely heard it mentioned, picked up on phrases, because we’re together, we live together, and I think they know. But I’m not sure if they really understand what cancer is.* (Patient 4)
95	*He came home yesterday with a huge drawing, a giant heart full of colour, and I said, “Hi, sweetie, thanks!”’You know, Mummy, I did it for you, I did it for when you were in hospital all day.’* (Patient 5)
96	*When we got home, we had to tell the kids that their dad had to have an operation and that he would no longer be able to breathe through his nose and mouth, but through a hole in his neck. It wasn’t easy. It was a bit of a struggle, to be honest. We tried to do it together, we tried to say it together, but then it was easier than usual, in the sense that the children weren’t upset at all. We were more upset by the news, but I guess it was also everything that had happened before, everything that had happened in the previous year.* (Patient 9)
97	*My son’s not the social type. He’s got a few mates, but he’s pretty shy and quiet. He had become really sad, always keeping to himself, much more withdrawn than usual.* (Patient 8)

**Uncertainty about what children have understood.** Many comments mention that parents did not know exactly what their children understood about the illness and treatments. Some parents tried to pick up “signals” from their children’s understanding from their behavior because they found it difficult to introduce the subject themselves and communicate effectively. Sometimes these “signals” were easy to interpret, for example when a child calmly explained to their teacher why it was important to take care of their sick mother (ref 86). At other times, it was more difficult for parents to understand what their children have internalized. Examples include a child reproaching their mother for spending a lot of time in bed (ref 87) and a teenager expressing concern about the effect of CT scans on their mother’s mood (ref 88).

This was mainly an issue for pre-school and school-age children, who did not primarily use words to communicate and understand their sick parent’s condition, but rather perceive the “presence” of the illness through changes in the family routine (refs 89–91).

Regardless of the age, picking up on signals was even more difficult when there was an implicit agreement of “unspoken rules.” Parents who reported having acted as if “nothing was wrong” with regard to their illness also described a similar pattern in their children. Although children appeared to perceive that something about their parents’ health was not well, they, likewise their parents, behaved as if nothing was wrong (ref 92–93). Some parents said that their children “sensed” that something was wrong (ref 94).

This pattern of communication continued until the children felt ready to give their parents the “green light” to talk, letting them know that they were comfortable with opening up (ref 95). In these cases, parents were amazed by their children’s level of understanding and processing.

**Similarities in communication styles between parents and children.** In this respect, the lack of awareness among the interviewed parents regarding their children adopting their own communication styles is of interest. If openness to dialogue and communication about the illness was present among parents, the same was true for their children. Conversely, if parents struggled to communicate about the disease, their children also found it difficult (refs 5 and 6).

Parents also found it hard to understand their children’s emotions, often seeing them as a “black box” that could not be accessed (ref 97). Indeed, no instances were found where a parent described their child as calm, restless or irritable when discussing the illness or treatment.

### Needs

We identified 57 needs, which were grouped into three macro-categories that were often not mutually exclusive: existential needs, support needs, and needs related to continuing to be and act as parents (see Table 8). These needs were derived from the themes presented above. For this reason, same quotations will not be shown as already presented.

**Table 8.** List of needs expressed by parents, divided by macro-areas

I need…	Existential needs	Support needs	Needs related to continuing to be and act as parents
my child to respect my way and timing of communicating the illness	X		
to experience the illness in a family context of harmony	X		
certainty about the progression of my illness	X		
to have some time in the day just for myself	X		
to have personal space within my daily life	X		
certainty about the outcome of my treatments	X		
to know why the cancer happened to me	X		
to be able to prevent any complications of my condition	X		
to face things as they come, day by day	X		
to be able to be at peace	X		
to find motivation to face the difficulties of daily life	X		
to return to normality	X		
to feel capable of being a good parent	X		X
to pass on skills and experiences to my child so they can face the future if I’m no longer there	X		X
to accept my health condition before communicating it to my child	X		X
to know that one day my partner and child will manage even without me	X		X
to know that I haven’t passed the illness on to my child	X		X
my suffering to be acknowledged by those close to me	X	X	
support from the healthcare professionals who are helping me return to daily life		X	
support from those close to me in returning to daily life		X	
healthcare professionals to consider my child’s age when giving me advice on how to communicate the illness		X	
my child to be involved in my treatment process		X	
my partner to understand my illness-related needs		X	
those close to me to understand my needs		X	
those close to me to understand the struggle resulting from my health condition		X	
(For caregivers only) my partner to communicate their illness to my child		X	
(For caregivers only) healthcare professionals to communicate the diagnosis/outcome of treatments to me first		X	
healthcare professionals following me to be fully aware of my medical history		X	
more information about my condition		X	
to be informed about the progress of research related to my condition		X	
a healthcare professional who can be a point of reference for me		X	
healthcare professionals following me to be coordinated with each other		X	
a healthcare professional to continuously monitor my child’s behaviour		X	
a healthcare professional to continuously monitor my child’s emotional state		X	
structured psychological support as part of my cancer care		X	
healthcare professionals following me to also monitor my child in cancer prevention		X	
to be able to get appointments for visits and exams quickly		X	
emotional support from those close to me		X	
healthcare professionals to answer my questions		X	
more information about prevention options for my child		X	
to know how much my child should know about my condition		X	
to be able to have visits and exams at an affordable cost		X	
to be able to have visits and exams at a location near my home		X	
my child’s teachers to agree to meet with me online		X	
my employer to understand the strain of my situation		X	
my colleagues to understand the strain of my situation		X	
psychological support		X	
psychological support for my whole family		X	
to understand how and what to say to my child about my illness/treatments I am undergoing		X	X
to be able to show my child that I can manage			X
to be able to show my child that I am okay			X
to see my child at peace regarding my condition			X
to be able to communicate to my child that his/her life must go on, even if I’m no longer here one day			X
to continue being a good parent despite the effects of the illness/treatments			X
to continue ensuring my child experiences “normality”			X
to have communicated my illness to my child in a sufficiently “gentle” way			X
to ensure I haven’t caused trauma to my child due to my illness			X

The needs that defined as “existential” were primarily related to the survival of parent as an individual. These included needs for parents to know the outcome of treatments or the progression of the illness, to understand their own struggles, to have their time and space respected by those around them and to be sure that, should they die, their family would be able to carry on functioning. Support needs varied and includedcare for themselves (e.g. physical and emotional support from loved ones and psychological support from professionals), as well as care and support for their children and, more broadly, the whole family. Many of these needs were related to improving the efficiency of the National Health Service and patient-centered care.

Finally, needs related to continuing to be and act as a parent included providing their children with a “normal” daily life, appearing strong and healthy and communicating effectively about their illness.

## Discussion

This study has highlighted the many challenges faced by parents affected by cancer. The two biggest challenges reported by the interviewed parents were managing the complexities of daily life and communicating the illness to their children.

Parents who experienced these challenges often felt concerned, guilty, lonely and afraid of experiencing a physical and/or emotional “breakdown.” Guilt related to both worrying that, one day, their children will not be able to live a “normal” life and not having taught their children to be more independent in case they die. Guilt also emerged in Schiena’s study (2019), where it was linked to striking the right balance between caring for oneself and caring for one’s children. In the same study, some parents also expressed this feeling when they had to rely on the community for help with their children’s daily lives, such as asking other parents to take their children to school or to cook for them, when support from family members was unavailable. These aspects did not emerge from this study’s interviews, possibly because, in Italy, grandparents often serve as the primary source of support for parents with young children (Zamberletti et al. 2018).

Other studies have emphasized the numerous concerns of parents with cancer, highlighting the need to ensure continuity and a sense of “normality” for their children, and the desire to continue being “good parents” (Semple and McCance 2010; Schiena et al. 2019). In addition to the challenges of being a mother with lymphoma, Elmberger et al.’s study (Elmberger et al. 2005) also highlighted some positive aspects identified by the women involved, such as their children becoming more responsible for household tasks like cooking and cleaning. By contrast, in the present study, mothers expressed strong concerns about not having made this transition. Despite social changes in Italy in recent decades, the traditional view of women as the “heart of the home” remains deeply rooted (Scabini and Iafrate 2019), which may explain their worry about not adequately “preparing” their children for their possible death.

The results of the present study reflect those of previous research highlighting the difficulties parents face in knowing when and how to talk to their children about the illness (Semple and McCance 2010). Semple et al.’s literature review emphasized the significant challenges parents face when communicating about the illness. They noted that effective decision-making is essential, particularly with regard to when to start the conversation, which words to use and how much information to share.

Among participants, there was a widespread belief that children were too young to understand their parents’ illness, or that discussing it might cause psychological or behavioral problems. For this reason, parents often believed that their children should know as little as possible about their condition. References to the illness were frequently avoided and the choice of terminology was deliberate (for example, words such as “cancer” and “metastasis” were avoided). Parents also tended to hide moments of vulnerability; for instance, they tried to conceal emotions such as despair and fear, as well as the physical fatigue caused by the illness and its treatments. It is essential to study beliefs because, not only do they refer to individuals’ perceptions of reality, but they form a crucial part of how people interpret experiences. In this sense, this study’s findings show that beliefs are closely linked to communication strategies. From an intervention perspective, the present study suggests that knowing and understanding parents’ beliefs is crucial in order to work on communication strategies with their children.

Although experts have long emphasized the importance of speaking to children about illness and death, this remains a significant challenge for parents. Developmental psychologists agree that most children aged 5–8 are capable of developing a “mature” understanding of death, meaning they grasp its irreversibility, the fact that the body no longer functions, and how people can die (Hunter and Smith 2008). Christ (2000) notes that a child’s understanding of death varies based on their experiences, as well as their cognitive, emotional, and social capacities. In this sense, the literature consistently stresses the importance of being open and honest in a way that matches the child’s social, cognitive and emotional development (Monroe and Kraus 2005). Even for adolescents, clear and empathetic communication from parents helps create a sense of predictability, helping them to understand what to do and when to do it (Goethals et al. 2019).

Another belief concerned the perceived usefulness of healthcare professionals in helping communicate the illness to children. Some studies reported that parents do not feel adequately supported by healthcare professionals when it comes to explaining the illness to their children (Semple and McCance 2010). This study also reveals the belief that healthcare professionals were not the “right people” to support parents in this process. The underlying idea appears to be that professionals were perceived as unable to provide personalized support – that is, support that takes into account the child’s age and level of understanding. There seems to be a certain distrust of the abilities of those responsible for this task, which contributes to unspoken issues.

The practice of not communicating certain aspects of the illness to children – or not communicating at all – leads to the added burden of uncertainty about children’s actual understanding. This uncertainty was sometimes not resolved until the children themselves made it clear to their parents, either directly or indirectly, that they have understood the real situation. This aspect has not yet been explored, but it deserves attention as it can create spirals of “unspoken” issues that may further burden parents and affect children’s adjustment.

Another noteworthy finding of the present study is that there was a “mirroring” of communication styles between parents and their children in interviewees’ quotations, although they seem unaware of this. Some parents expressed surprise when describing how their children “don’t talk much” or struggle to express their feelings, yet they describe themselves in the same way. Similarly, other parents described their children as open to dialogue and eager to ask questions about the illness; these parents also reported using the same communication approach. The recent systematic review by Mckeown et al. (2025) highlighted that children may decide to avoid communication to protect their parents as well, this constrained pattern of communication with children’s parents appears reciprocal.

While the latter study represents the only reference found in the existing literature regarding the transmission of communication styles in the context of cancer-related communication, some additional studies highlighted the relationship between communication style and children’s adaptation to metabolic diseases. These studies have shown that an “open,” collaborative and empathetic parenting style improves metabolic and psychosocial outcomes (e.g. health-related quality of life and motivation) in children, in contrast to more intrusive, controlling, authoritarian or avoidant styles (Shorer et al. 2011; Young et al. 2014; Goethals et al. 2019).

The needs of the interviewed parents were numerous and varied, and many of them were unmet. While some needs, such as receiving a definite prognosis, were legitimate but could not always be met, others could be addressed, such as improving the efficiency of the National Health System and making psychological support a standard care option for patients and their families. The parents in this study shared several needs that have already been identified in the general oncology population (such as “existential” and support-related needs), as they have been found in studies conducted within the same hospital (Tamburini et al. 2003; Alfieri et al. 2022, 2025). However, others needs were specific to being a parent and to having children at a particular stage in their life cycle. Understanding these parents’ needs is a starting point for addressing the current gap in the literature and for considering how to respond to these needs. For this reason, a preliminary draft of a needs checklist has been developed, which future studies could use to create a validated tool. Once its psychometric properties have been tested, the checklist could be used widely in clinical settings as a practical screening tool to identify needs at key stages of parents’ care journeys. It could, for instance, be structured as a list of yes/no items or as a Likert scale (e.g. from 1 = “not at all” to 5 = “very much”). Once finalized, this checklist could be administered alongside a quality of life (QoL) instrument to systematically monitor needs during key phases of the illness trajectory (e.g. diagnosis, treatment, follow-up and end of life) (Brunelli et al. 2022).

Engaging in a process of reflexivity, we acknowledge that our analysis is informed by our way of being and seeing the world. In particular, we would like to highlight the characteristics of the three researchers who carried out the analyses. Firstly, they were three women, who may have had a different perspective from that of men when it comes to constructing meanings. Secondly, two of the researchers have young children, which has led to a strong emotional involvement in the issues addressed and in the “weight” attributed to the meanings constructed. We explain these aspects far from considering them biases, but because we are aware that they are important aspects of the narrative we have created, as required by the approach used (Braun and Clarke 2019, 2024).

### Study limitations

This study has certain limitations that should be taken into consideration. Firstly, participants were recruited from a single Comprehensive Cancer Centre. While this enables targeted interventions to be developed to support patients within the same hospital, it also limits the generalizability of the findings. Furthermore, it is worth considering that the majority of the parents involved were engaged in psychological support care pathway. This may have increased the awareness of their own characteristics, abilities and needs, resulting in a bias. Secondly, as no specific analysis was conducted regarding the age of the children, differences likely exist in parenting approaches or communication styles depending on whether the children are pre-school, school-age or adolescents. Third, a qualitative study does not allow us to “prioritize” needs: currently, there is a snapshot of all the needs expressed by parents, but it is not known which of these are “vital” and which are not.

### Clinical implications

The results of this study have several important practical applications. Communicating with children is a particularly sensitive and crucial aspect of a parent’s experience of cancer, and healthcare professionals should recognize this rather than leaving it solely to the parent. This is essential to prevent feelings of concern, guilt and loneliness from escalating to the point of emotional collapse. As previous studies have highlighted (Schiena et al. 2019), oncology services should routinely record whether the patient has underage children. Too often information is limited to the patient’s health status without considering what elements may represent an additional burden or, conversely, a source of support. Secondly, healthcare professionals must be adequately trained to manage issues arising from interactions between parents with cancer and their children under the age of 18. This involves offering appropriate support to help parents improve their communication skills and assisting children in identifying and coping with distress and stress related to their parents’ illness. These interventions aim to enhance the parents’ quality of life and prevent or alleviate psychological distress among their children. Support is needed throughout all phases of the illness, including diagnosis, treatment and follow-up, as well as the terminal and end-of-life stages (Christ 2000).

## Supporting information

10.1017/S1478951525101272.sm001 Alfieri et al. supplementary material Alfieri et al. supplementary material

## Data Availability

The data that support the findings of this study are available from the corresponding author upon reasonable request.

## Supplementary material

The supplementary material for this article can be found at https://doi.org/10.1017/S1478951525101272
