# Supplementary material for: Communicating cancer to children: Strategies and needs of parents with cancer. A qualitative study
Source: Palliat Support Care. 2026 Jan 8;24:e23. doi: 10.1017/S1478951525101272 (PMC13166336; doi:10.1017/S1478951525101272)
Supplement: Alfieri et al. supplementary material [file S1478951525101272sup001.docx]

Appendix 1: Interview Outline

1. What do your children know about your illness?

a. How did they find out?

b. How did you figure out what to tell them?

2. What do you think they understood?

3. What was the most difficult part of talking to your children?

4. Did you consult any experts on how to approach this topic with your children?

5. What are the needs of a parent with cancer who has children of the same age as yours?
